# A Rare Case of Vasospastic Angina Presenting with Inferior Lead ST-segment Elevation and Ventricular Fibrillation in the Absence of Coronary Obstruction: A Case Report

**DOI:** 10.7759/cureus.6332

**Published:** 2019-12-09

**Authors:** Derman Ozdemir, Joshi Kishor, Julia M Hall, Hal Chadow, Shahrokh E Rafii

**Affiliations:** 1 Internal Medicine, Saba University School of Medicine, Saba, NLD; 2 Internal Medicine, Brookdale University Hospital and Medical Center, Brooklyn, USA; 3 Cardiology, Brookdale University Hospital and Medical Center, Brooklyn, USA

**Keywords:** vasospastic angina, coronary angiogram, ventricular fibrillation, non-obstructive coronary artery disease, st-segment elevation, prinzmetal's angina

## Abstract

Vasospastic angina (VSA) is a variant form of angina pectoris, which occurs at night or at rest, with transient electrocardiogram modifications and preserved exercise capacity. Its association with stable angina, sudden cardiac death, acute coronary syndrome, arrhythmia, and syncope has previously been established. Its presentation can occur with or without existing coronary artery disease and may present with focal or diffuse alteration and dysfunction of the coronary vasculature. VSA diagnosis involves patient response to nitrates, transient ischemic electrocardiogram (ECG) changes, and coronary artery spasms. The mechanisms proposed to constitute the substrate for susceptibility to VSA include vascular smooth muscle cell hyperreactivity, endothelial dysfunction, magnesium deficiency, low-grade inflammation, altered autonomic nervous system response, hypothyroidism, and oxidative stress. Herein, we present the rare case of a patient with ST-segment elevation in the inferior leads, increased troponin, and an episode of ventricular fibrillation initially thought to be due to lateral wall ST-elevation myocardial infarction (STEMI), although it was revealed to be vasospastic angina. We will also review the literature. Vasospastic angina remains underdiagnosed and a timely diagnosis is crucial to prevent major cardiac events. In patients with diffuse ST-segment elevation on ECG (independently of angiographic findings), VSA should be considered as one of the differential diagnoses and treated if found to be the cause of pathological changes.

## Introduction

Vasospastic angina (VSA), more commonly known as Prinzmetal or variant angina, is classified as spontaneous episodes of angina pectoris produced by coronary epicardial vasospasm, as well as those induced during provocative coronary vasospasm testing and coronary microvascular dysfunction due to microvascular spasm [1]. The hallmark feature of VSA is angina at night or at rest which frequently lasts longer than the usual episode of angina and is often accompanied by ST-segment elevation that is transient and reversed in minutes after the administration of nitroglycerin (NTG). However, VSA has a range of significant clinical symptoms ranging from stable angina to arrhythmias to sudden cardiac death. An adapted version of the diagnostic criteria for VSA, as proposed by the Coronary Vasomotion Disorders International Study Group (COVADIS) [2], highlights three key factors:

1) Nitrate-responsive angina during the spontaneous episode with at least one of the following:

*Rest angina, especially between night and early morning;

*Marked diurnal variation in exercise tolerance, reduced in the morning;

*Hyperventilation can precipitate an episode;

*Calcium channel blockers (but not beta-blockers) suppress episodes.

2) Transient ischemic electrocardiogram (ECG) changes - during the spontaneous episode, including any of the following in at least two contiguous leads: ST-segment elevation ≥ 0.1 mV, ST-segment depression ≥ 0.1 mV, and new negative U waves.

3) Coronary artery spasm defined as transient total or subtotal coronary artery occlusion (> 90% constriction) with angina and ischemic ECG changes either spontaneously or in response to a provocative stimulus (typically, acetylcholine, ergonovine, or hyperventilation).

The importance of accurately diagnosing VSA in patients with ST-segment elevation is crucial to prevent acute major cardiac events and long-term complications associated with VSA. Herein, we describe the case of a 66-year-old male presenting with an ECG finding of inferior wall ST-segment elevation myocardial infarction (STEMI) most likely secondary to symptomatic VSA, as he was found to have no coronary obstruction and met the diagnostic criteria for VSA as proposed by COVADIS.

## Case presentation

A 66-year-old morbidly obese African-American male called emergency medical services (EMS) with a chief complaint of worsening substernal chest pain over the course of three hours. His past medical history included hypertension, type 2 diabetes mellitus (T2DM), Stage 3 chronic kidney disease, obstructive sleep apnea, and angina. The patient reported having a nuclear stress test (NST) in 2017 which showed “mild to moderate inferoposterior segmental perfusion defect” and premature ventricular contractions (PVCs); however, an NST one month before presentation did not show any abnormalities. Normally compliant with his medications and follow-up visits regarding his health, he was incarcerated and was not able to take his medication regimen of aspirin (81 mg), isosorbide dinitrate (30 mg), and as needed nitroglycerin tablets for two days. He denied any history of smoking, alcohol, or drug use. He also denied a history of shortness of breath, palpitations, sweating, dizziness, nausea, vomiting, weight loss, or fever. His weight was noted to be 123 kg with a body mass index (BMI) of 43.1 kg/m^2^. On EMS arrival, his systolic blood pressure was 150 - 160 mmHg with his heart rate at 70 - 80 beats per minute. The patient reported relief of his symptoms after he was given nitroglycerin spray (0.4 mg) and aspirin (162 mg). An ECG done after treatment showed no acute ST-T changes and he was brought to the emergency department (ED) without any incident. In the emergency department (ED), the patient was awake, alert, oriented, and in slight distress with vital signs within normal limits. On physical examination, he was noted to be obese with no evidence of respiratory distress and was found to have mild tenderness upon palpation of the chest along the sternum, but the rest of the exam was unremarkable. His lungs were clear to auscultation bilaterally. Abdominal and neurological examinations were unremarkable. Initial laboratory values are summarized in Table 1.

**Table 1:** Initial Laboratory Values PBNP: pro-brain natriuretic peptide

Laboratory Test	Results
Hemoglobin {g/dl}	16.3
Hematocrit (%)	46.0
White blood cells (K/ul)	5.4
Sodium (mmol/L)	138
Potassium (mmol/L)	5.1
Chloride (mmol/L)	102
Magnesium (mg/dL)	1.9
Blood urea nitrogen (mg/dl)	26
Creatinine (mg/dl)	1.43
Glucose (mg/dl)	228
Hemoglobin A1C (%)	10.3
PBNP (Pg/mL)	56.8
Troponin (ng/mL)	0.022
Drug screen	Negative

**Figure 1:**
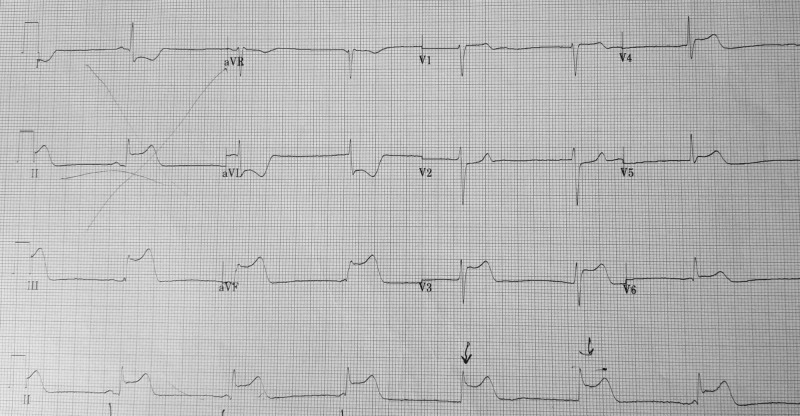
Initial electrocardiogram (ECG) on patient presentation ST-segment elevation in the inferior leads (II, III, aVF)

**Figure 2:**
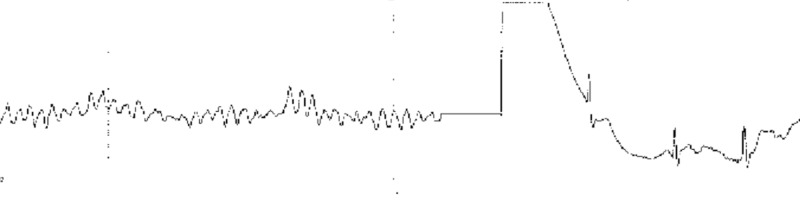
Ventricular fibrillation before cardioversion

**Figure 3:**
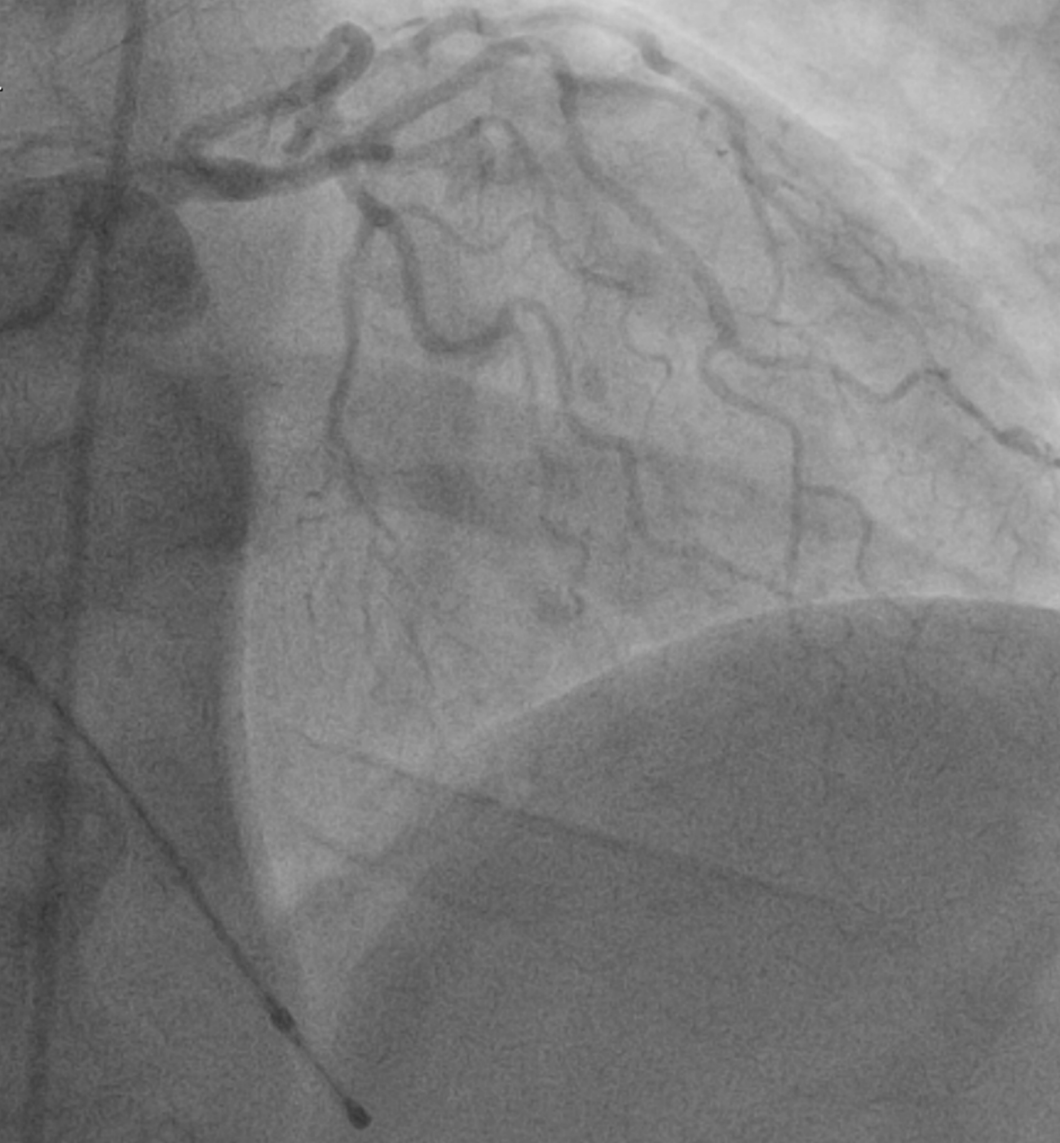
Left coronary artery Angiography showing coronary spasms and subsequent blood flow distribution of the left coronary artery before nitroglycerin

**Figure 4:**
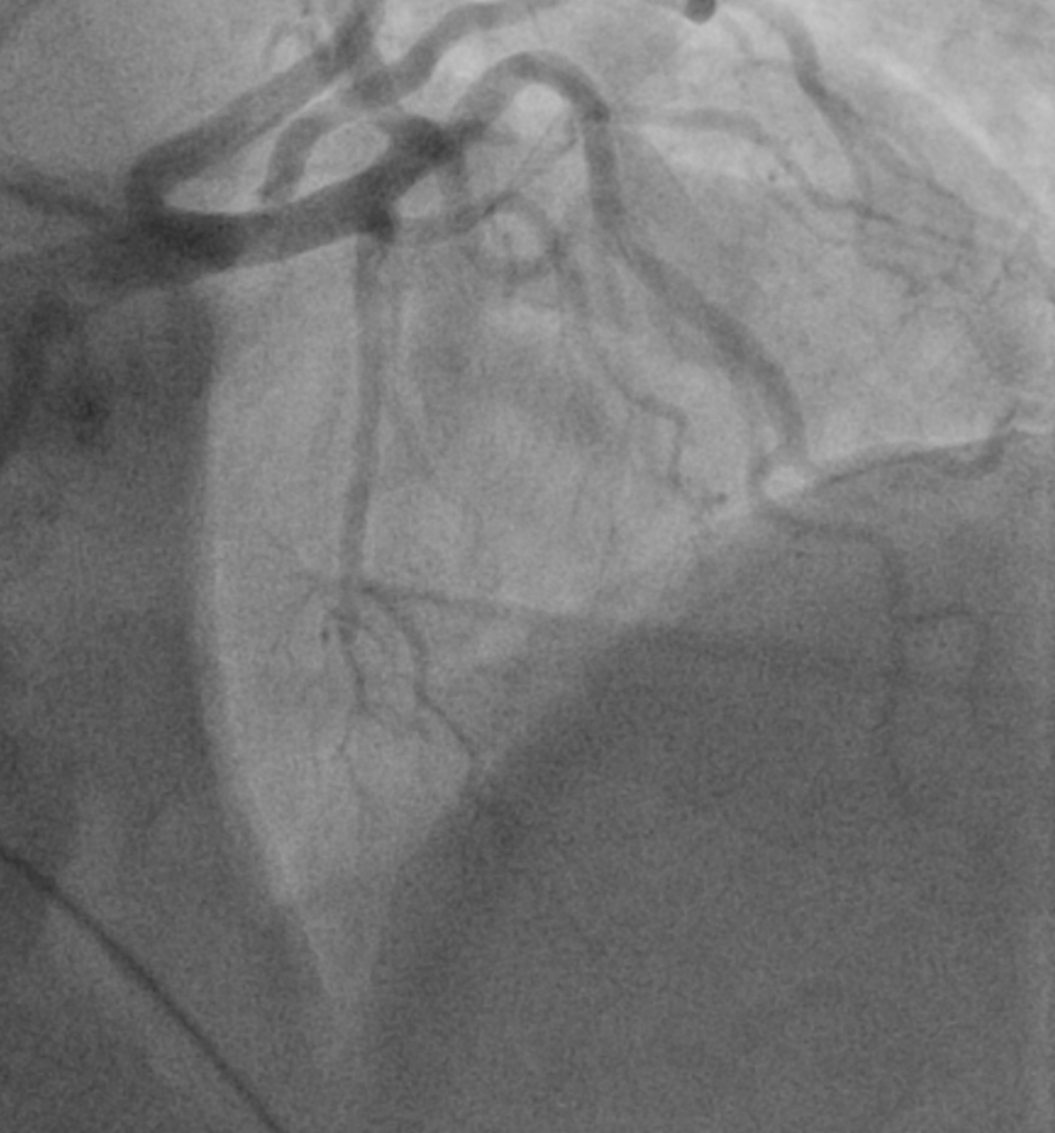
Left coronary artery Angiography showing resolution of coronary spasms and subsequent increased blood flow distribution of the left coronary artery after nitroglycerin

**Figure 5:**
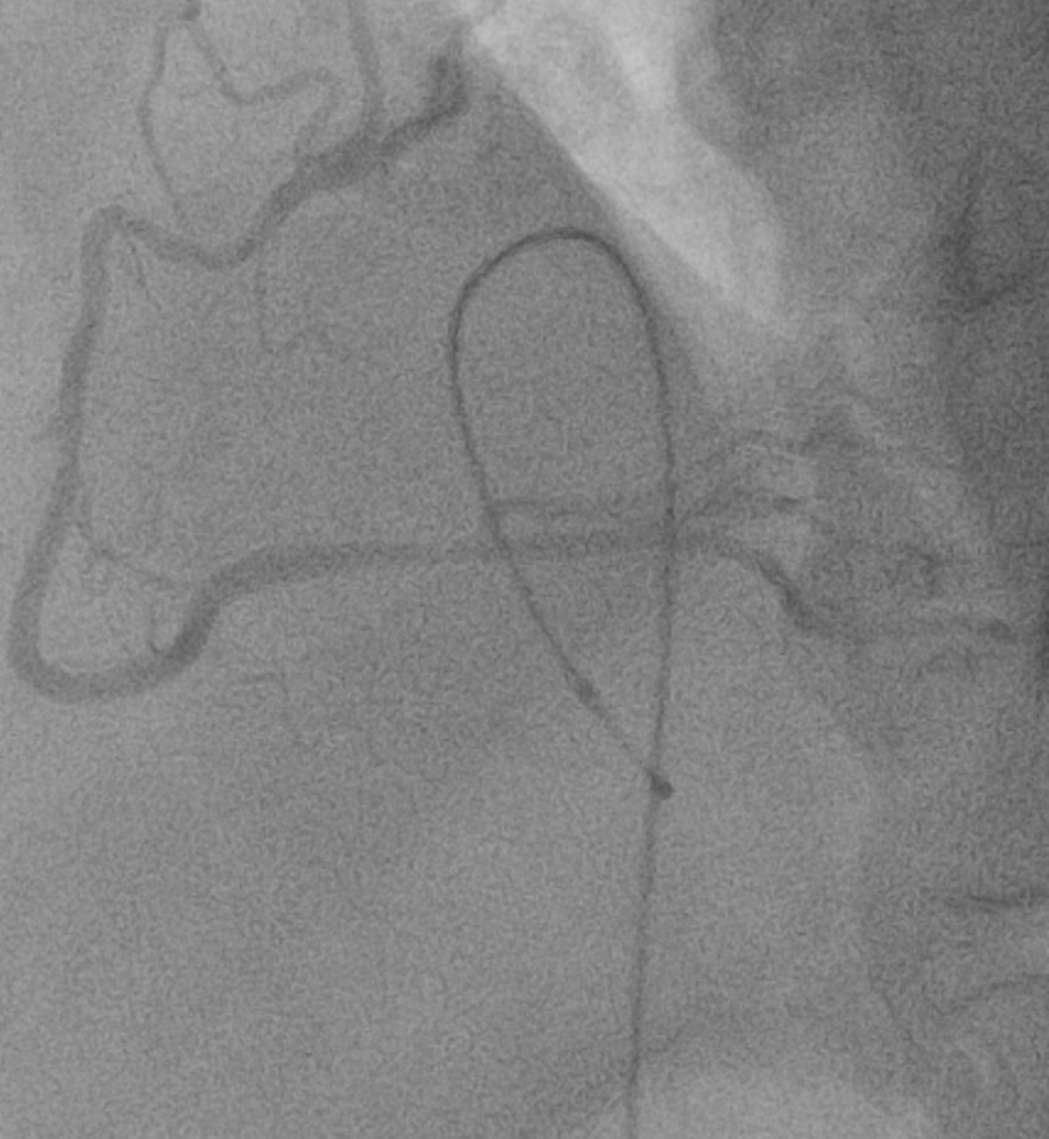
Right coronary artery Angiography showing coronary spasms and subsequent blood flow distribution of the right coronary artery before nitroglycerin

**Figure 6:**
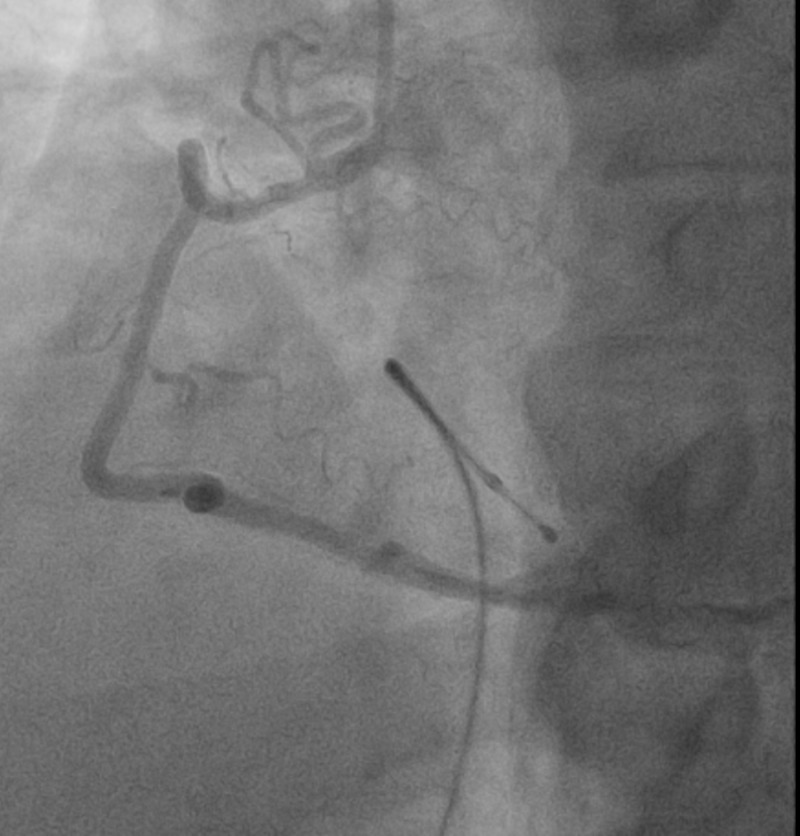
Right coronary artery Angiography showing resolution of coronary spasms and subsequent increased blood flow distribution of the right coronary artery after nitroglycerin

**Figure 7:**
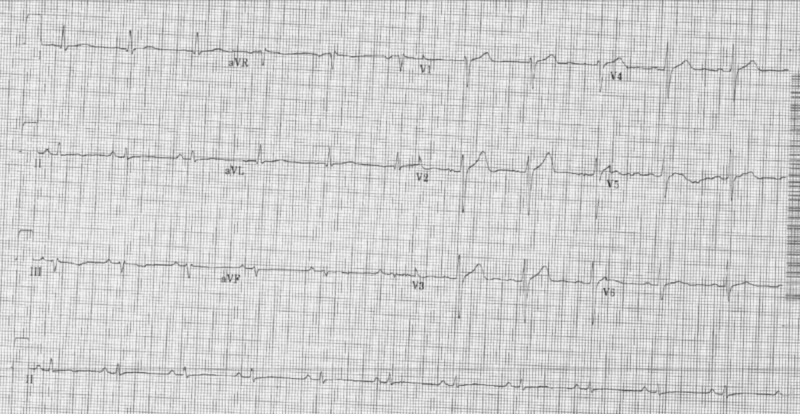
Electrocardiogram (ECG) in the coronary care unit Normalized ECG with appropriate ST-segments and rate

There were no significant events upon initial presentation; however, one hour later, the patient then began to sweat profusely and reported to be in worse distress. A repeat ECG revealed sinus bradycardia and ST-segment elevation in the inferior wall leads (II, III, aVF) (Figure 1). The patient was loaded with aspirin (162 mg), ticagrelor (180 mg), and atorvastatin (80 mg), per the acute coronary syndrome protocol. The patient was emergently taken for coronary catheterization and developed ventricular fibrillation (VF). Cardiopulmonary resuscitation (CPR) was initiated and defibrillation with 200 joules was administered on the stretcher which was effective in returning the biphasic rhythm to sinus rhythm (Figure 2). The patient became responsive and was placed on the catheterization table before the coronary catheterization was continued and completed through a right femoral artery approach. Coronary angiography revealed normal ejection fraction (EF) of 60% with non-obstructive coronary artery disease (CAD), along with diffuse spasms of the coronary vessels. Nitroglycerin was administered during the catheterization and the patient responded with a resolution of coronary spasms and increased blood flow prompting no further intervention and transfer of the patient to the coronary care unit (CCU) (Figures 3-6). In the CCU, the patient’s blood pressure increased to 205/100 mmHg and was subsequently controlled with a nitroglycerin drip (20 mcg/kg). The only significant event in the CCU was an initial increase in troponin levels to 0.266 from 0.022 ng/mL. Both troponin levels and ECGs normalized on hospital day 3 and the patient remained stable until discharge (Figure 7). Implantable cardioverter-defibrillator (ICD) implantation was conducted prophylactically once the patient became hemodynamically stable due to his episode of VF and the high risk of recurrence that is associated with VF. The patient was discharged on hospital day 6 with atorvastatin (80 mg), isosorbide mononitrate (30 mg), nifedipine (60 mg), and furosemide (40 mg).

## Discussion

Vasospastic angina refers to attacks caused by vasospasm of one or more coronary arteries. Even though its exact etiology is still unknown, mechanisms, such as vascular smooth muscle cell hyperreactivity, underlying atherosclerosis, endothelial dysfunction, magnesium deficiency, low-grade inflammation, and oxidative stress, have been noted in the literature [3].

Pathophysiology

The variant form of angina was first described in 1959 by Prinzmetal et al. [4] who used this term to indicate that angina attacks, unlike the most common form of effort angina, occurred at rest and were associated with ST-segment elevation (rather than ST-segment depression) on the ECG. Prinzmetal et al. also reported that ST deviation is related largely to a change in electrolytes. This change in intra- and extracellular electrolyte balance occurs in ischemic heart disease, as well as in a wide variety of non-cardiac conditions [5]. In addition to precipitating factors listed previously, alkalosis secondary to hyperventilation, cocaine intoxication, mental stress, the use of pharmacological molecules that alter autonomic nervous system response, such as sympathomimetic agents (adrenaline and noradrenaline), para-sympathomimetic agents, ergot alkaloids (ergonovine and ergotamine), and beta-blockers, can also trigger vasospasm. Beta-blockers inhibit beta-adrenergic receptor-mediated coronary vasoconstriction and increase vascular permeability to calcium by unmasking alpha-adrenergic receptor-mediated coronary vasoconstriction [6]. In the presented case, even though VSA was most likely secondary to mental stress from incarceration and exacerbated by not having access to his medications, beta-blockers were avoided to decrease the likelihood of adverse effects.

Epidemiology

Vasospastic angina rates in the Western countries had been steadily decreasing most likely secondary to more effective and more widely used pharmacological interventions for chest pain and hypertension (calcium channel blockers (CCBs), nitrates) and decreased interest from physicians in provocation testing and diagnosis [7]. However, more recently, in a large national analysis, there has been an increase in the prevalence of hospitalizations with Prinzmetal angina. Older age, heart failure, chronic kidney disease, chronic liver disease, and acute myocardial infarction were predictors of higher mortality among patients with Prinzmetal angina. Patients with Prinzmetal angina who developed acute myocardial infarction had more favorable outcomes compared with myocardial infarction without Prinzmetal angina [8]. The prevalence of VSA remains largely unknown but studies show that it constitutes between 3% and 95% of all myocardial infarction with non-obstructive coronary atherosclerosis (MINOCA) patients depending on the stimuli used to trigger vasospasm, definitions of vasospasm, and ethnic background [9]. However, in Eastern nations (Japan and Korea), provocation testing is still being employed routinely. A recent survey showed that coronary spasm was documented in 921 (40.9%) of the 2,251 consecutive patients with angina pectoris who underwent coronary angiography [10]. Prognostic studies from the 1980s, performed on hundreds of VSA patients, demonstrated that a five-year survival rate free from death or myocardial infarction were 77% to 97% and 60% to 83%, respectively [11]. However, in a recent study of 245 Japanese patients with VSA, the survival rates at 1, 3, 5 and 10 years were 98%, 97%, 97%, and 93%, respectively. Survival rates without myocardial infarction at 1, 3, 5 and 10 years were 86%, 85%, 83% and 81%, respectively [12]. The high prevalence and incidence of VSA have led to the establishment of the Japanese Coronary Spasm Association (JCSA) risk score to provide risk assessment and prognosis for VSA patients [13]. The JCSA risk score is currently not used in Western nations; however, VSA diagnosis remains crucial to not only prevent associated major cardiac events (myocardial infarction (MI), syncope, arrhythmia, sudden cardiac death) but to prevent future adverse events with the use of selected pharmacotherapy and electrophysiological intervention [14].

Management

As with other diseases that are multifactorial and without exact etiology, management of VSA begins with lifestyle changes but pharmacological therapies directed at interrupting biochemical causes and avoiding precipitating factors should be implemented [3]. In the presented case, the patient was following up with a bariatric surgery team for his weight and was on daily nitrate therapy and nitroglycerin as needed. In terms of pharmacotherapy, Chahine et al. reported the effectiveness of CCBs (both dihydropyridine and non-dihydropyridine) in reducing angina frequency which is why calcium channels blockers are recommended as a first-line treatment in newly diagnosed VSA [15]. CCBs are effective therapies for VAS due to their mechanism of action which prevents vasoconstriction and promotes vasodilation in the coronary vasculature. Nitrates are another treatment option that dilates the coronary vessels and reduces ventricular filling pressures to decrease oxygen demand and ischemia. Extensive trials have demonstrated that nitrates are an effective treatment for reducing the frequency of angina in patients with VSA. Studies looked at statin drugs in conjunction with CCBs in preventing coronary artery vasospasm and found that the addition of fluvastatin to CCBs for six months significantly reduced acetylcholine-provoked spasm in patients with VSA without obstructive coronary artery disease [16-17]. There was a correlation between starting statin drug therapy and reduced long-term cardiovascular events in patients with VSA without obstructive coronary artery disease. Rho-kinase, which is known to reduce the contraction of the vascular smooth muscle cell (VSMC), was found to significantly reduce acetylcholine-induced vasoconstriction in patients where coronary vasospasm was induced by acetylcholine and prevented the occurrence of chest pain and ischemic electrocardiogram changes [18].

Lastly, the associated comorbidity of sudden cardiac death, as a result of ventricular arrhythmias, has shown a positive response to ICDs. Implantable cardioverter-defibrillators (ICD) have been shown to prevent sudden cardiac death. Even though the data regarding the use of ICD therapy remains an unexplored field, a retrospective study showed appropriate ICD shocks in 24.1% of patients with vasospastic angina who underwent ICD implantation for secondary prevention [19]. They were successfully resuscitated from second ventricular tachycardia, ventricular fibrillation, or pulseless electrical activity. Thus, in patients with life-threatening ventricular arrhythmias as a result of VSA, an ICD should be considered as there is a high risk of recurrence despite optimal medical management [20].

## Conclusions

The importance of accurately diagnosing VSA in patients with transient ST segment elevation on ECG with normal or near normal coronary angiograms is crucial to not only prevent major cardiac events but to prevent future adverse events with the use of appropriate pharmacotherapy and electrophysiological intervention. Even though vasospastic angina is rare in Western countries, it should not be overlooked in situations of chest pain and transient ST-segment elevation and the use of provocative testing to diagnose VSA should be considered more frequently. Patients should be treated with calcium channel blockers and the use of nitrates, statins, Rho-kinase inhibitors, and nicorandil should be discussed for optimal management of VSA. ICD implantation should become standard treatment for patients with VAS and history of arrhythmias due to the high risk of recurrence.
